# The Pt(*S*-pr-thiosal)2 and BCL1 Leukemia Lymphoma: Antitumor Activity In Vitro and In Vivo

**DOI:** 10.3390/ijms23158161

**Published:** 2022-07-24

**Authors:** Zana Besser Silconi, Vesna Rosic, Sasa Benazic, Gordana Radosavljevic, Marina Mijajlovic, Jelena Pantic, Zoran R. Ratkovic, Gordana Radic, Aleksandar Arsenijevic, Marija Milovanovic, Nebojsa Arsenijevic, Jelena Milovanovic

**Affiliations:** 1 Department of Cytology, Pula General Hospital, 52100 Pula, Croatia; zbsilconi@gmail.com; 2 Department of Histology and Embryology, Faculty of Medical Sciences, University of Kragujevac, 34000 Kragujevac, Serbia; vecarosic@gmail.com; 3 Department of Transfusiology, Pula General Hospital, 52100 Pula, Croatia; sbenazic@gmail.com; 4 Center for Molecular Medicine & Stem Cell Research, Faculty of Medical Sciences, University of Kragujevac, 34000 Kragujevac, Serbia; perungr@gmail.com (G.R.); panticjelena55@gmail.com (J.P.); aleksandar@medf.kg.ac.rs (A.A.); marijaposta@gmail.com (M.M.); arne@medf.kg.ac.rs (N.A.); 5 Department of Pharmacy, Faculty of Medical Sciences, University of Kragujevac, 34000 Kragujevac, Serbia; marina_kg87@yahoo.com (M.M.); vasic_gordana@yahoo.com (G.R.); 6 Department of Chemistry, Faculty of Science, University of Kragujevac, 34000 Kragujevac, Serbia; wor@kg.ac.rs

**Keywords:** BCL1, Pt(*S*-pr-thiosal)2 complex, antitumor activity, Bcl-2, STAT3

## Abstract

B cell malignancies are, despite the development of targeted therapy in a certain percentage of the patients still a chronic disease with relapses, requiring multiple lines of therapy. Regimens that include platinum-based drugs provide high response rates in different B cell lymphomas, high-risk chronic lymphocytic leukemia (CLL), and devastating complication of CLL, Richter’s syndrome. The aim of this study was to explore the potential antitumor activity of previously synthetized platinum(IV) complex with alkyl derivatives of thyosalicilc acid, PtCl2(*S*-pr-thiosal)2, toward murine BCL1 cells and to delineate possible mechanisms of action. The PtCl2(*S*-pr-thiosal)2 reduced the viability of BCL1 cells in vitro but also reduced the growth of metastases in the leukemia lymphoma model in BALB/c mice. PtCl2(*S*-pr-thiosal)2 induced apoptosis, inhibited proliferation of BCL1 cells, and induced cell cycle disturbance. Treatment of BCL1 cells with PtCl2(*S*-pr-thiosal)2 inhibited expression of cyclin D3 and cyclin E and enhanced expression of cyclin-dependent kinase inhibitors p16, p21, and p27 resulting in cell cycle arrest in the G1 phase, reduced the percentage of BCL1 cells in the S phase, and decreased expression of Ki-67. PtCl2(*S*-pr-thiosal)2 treatment reduced expression of phosphorylated STAT3 and downstream-regulated molecules associated with cancer stemness and proliferation, NANOG, cyclin D3, and c-Myc, and expression of phosphorylated NFκB in vitro and in vivo. In conclusion, PtCl2(*S*-pr-thiosal)2 reduces STAT3 and NFκB phosphorylation resulting in inhibition of BCL1 cell proliferation and the triggering of apoptotic cell death.

## 1. Introduction

The majority, about 95% of lymphomas diagnosed in about 20 cases per 100,000 people per year in Western countries are of B-cell origin [1]. Chronic lymphocytic leukemia (CLL), the most common leukemia in adults in Western countries, but very rare in the Far East, is also mostly always of B cell origin [2]. Inhibition of mitochondrial apoptosis is the key feature of all B cell lymphomas and also CLL [3]. Disrupted expression of BCL-2 protein contributes to the pathogenesis of almost all B cell lymphomas and leukemia [3,4]. Other anti-apoptotic proteins including MCL-1 are also involved in the pathogenesis of B cell NHL [5]. Aberrant expression of MCL-1 is partially due to constitutive activation of the STAT3 pathway in activated B-cell-like lymphoma [6]. The hallmark of some activated B-cell-like lymphomas and CLL is also constitutive activation of the NFκB pathway [7,8]. Stimulation through various surface receptors, including the B-cell receptor, results in the activation of the canonical NFκB pathway [9], and also in phosphorylation of STAT3 and activation of STAT3 signaling [10]. The use of novel approved targeted therapies in B cell malignancies that target different signaling pathways, has improved the prognosis of these patients [11]. However, combined regimens for chemoimmunotherapy resistant CLL, Richter transformation of CLL clone to diffuse large B-cell lymphoma or Hodgkin’s variant of Richter transformation, and some primary B lymphomas include platinum-based drugs that provide higher response rates in these patients [12]. Platinum-containing chemotherapeutics, even decades after the introduction of the first platinum-based drug, represent the most numerous group among drugs used in the treatment of various tumors. Therapeutic protocols in more than half of people with various tumors contain a platinum-based drug, indicating the unequivocal importance of the research into new metal complexes [13]. Conjugating natural products derived from different plants with transition metals is a strategy used to obtain complexes that exhibit less toxic effects [14]. Salicylates are natural substances firstly isolated from myrtle and then from different plants that exhibit different biological activities in vivo associated with a reduced risk of tumor development as indicated by the results of some epidemiological studies [15,16]. Salicylates manifest an apoptotic effect in tumor cells by regulating the PTEN/AKT/NF-κB/surviving signaling pathway [17], and possibly by inducing stress on the endoplasmatic reticulum [18]. Modification of salicylates [19] and conjugation of modified salicylates with different metals [20,21] resulted in compounds with stronger antitumor activity. We have previously synthesized and characterized platinum(IV) complexes with S-alkyl derivatives of thiosalicylic acid [19] and we have shown significant cytotoxic activity of these complexes toward murine leukemia lymphoma cell line (BCL1) and human B-prolymphocytic leukemia (JVM-13) [22]. Others also reported stronger antitumor activity of platinum complexes with non-steroidal anti-inflammatory drugs than cisplatin [23]. Complexes of S-alkyl derivatives of thiosalicylic acid with some other metals, copper and zinc, also have shown marked antitumor activity toward colorectal [20] and breast cancer [21] in vitro and in vivo. Copper(II) complexes exhibited pro-apoptotic and anti-proliferative effects, and induced cell cycle disturbances in colorectal cell lines [20]. Zn(*S*-pr-thiosal)2 complex induced apoptosis of murine breast cancer cell line, inhibited cell proliferation through G1/S cell cycle arrest, and markedly inhibited murine breast cancer growth [21]. This complex also decreased the expression of STAT3 in murine breast cancer cells and decreased the expression of STAT3 targeted genes [21]. The highest cytotoxic potential toward murine B cell leukemia lymphoma cell line, BCL1, showed platinum(IV) complex with S propyl thiosalicylic acid (named Pt(*S*-pr-thiosal)2 complex), similar to the activity of cisplatin, based on half maximal inhibitory concentration (IC50) values 2.47 ± 0.84 for Pt(*S*-pr-thiosal)2 versus 2.04 ± 0.79 for cisplatin [22]. Inhibition of STAT3 phosphorylation by different platinum complexes has been reported previously [24,25].

Here we have reported that complex PtCl2(*S*-pr-thiosal)2 reduced significantly the viability of BCL1 cells in vitro and also reduced the growth of metastases in the BCL1 leukemia lymphoma model in BALB/c mice. Treatment of BCL1 cells with PtCl2(*S*-pr-thiosal)2 inhibited the expression of cyclin D3 and cyclin E and enhanced the expression of cyclin-dependent kinase inhibitors p16, p21, and p27 resulting in cell cycle arrest in the G1 phase. A reduced percentage of BCL1 cells treated with PtCl2(*S*-pr-thiosal)2 in the S phase of the cell cycle was accompanied by a decreased percentage of Ki-67 positive cells, indicating anti-proliferative effects of the tested complex. Treatment with PtCl2(*S*-pr-thiosal)2 also reduced the expression of phosphorylated STAT3 and downstream-regulated molecules associated with cancer stemness and proliferation, NANOG, cyclin D3, and c-Myc, and the expression of phosphorylated NFκB in vitro and in vivo. In conclusion, PtCl2(*S*-pr-thiosal)2 reduced STAT3 and NFκB phosphorylation resulting in inhibition of cell proliferation and the triggering of apoptotic cell death.

## 2. Results

### 2.1. Pt(S-pr-thiosal)2 Complex Causes Caspase-Dependent Apoptotic Death of BCL1 Cells

**Figure 1:**
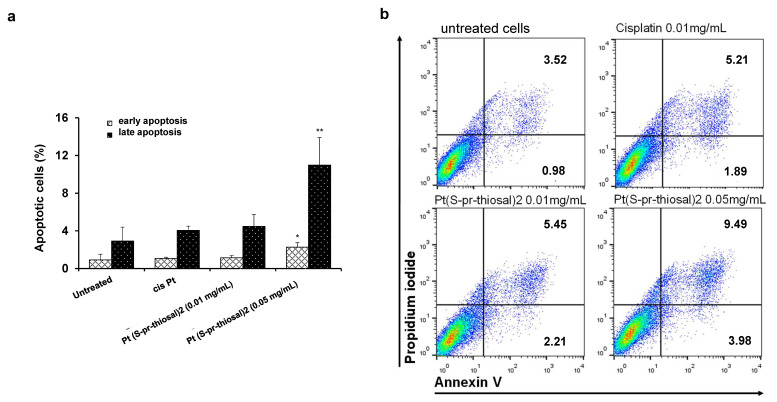
Pt(*S*-pr-thiosal)2 induces apoptotic death of BCL1 cells. (**a**) Results for the percentage of cells in early (annexin V+ propidium iodide-) and late (annexin V+ propidium iodide+) apoptosis detected by flow cytometry are shown as mean + SD for 3 independent experiments. The statistical significance of the difference in the percentage of apoptotic cells was determined by Student’s *t* test; * *p* < 0.05, ** *p* < 0.01 and indicates the difference in the percentage of BCL1 cells treated with platinum complex at 0.05 mg/mL and all three other groups of BCL1 cells (untreated; treated with cisplatin; and treated with platinum complex at 0.01 mg/mL). (**b**) Representative dot plots show the population of BCL1 cells in early and late apoptosis.

**Figure 2:**
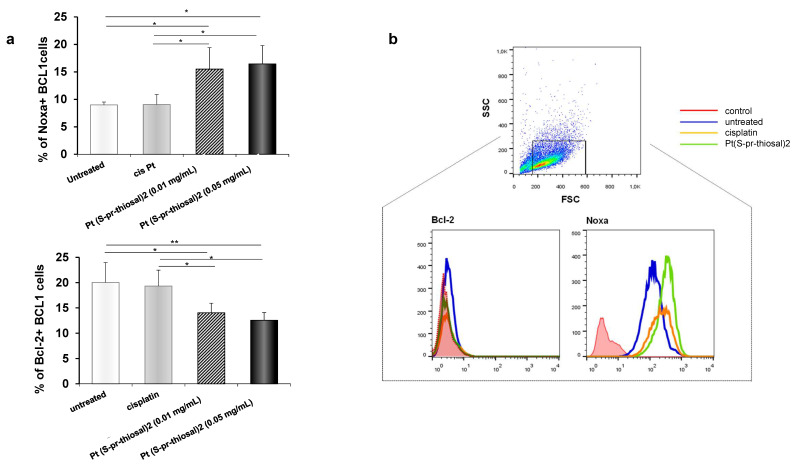

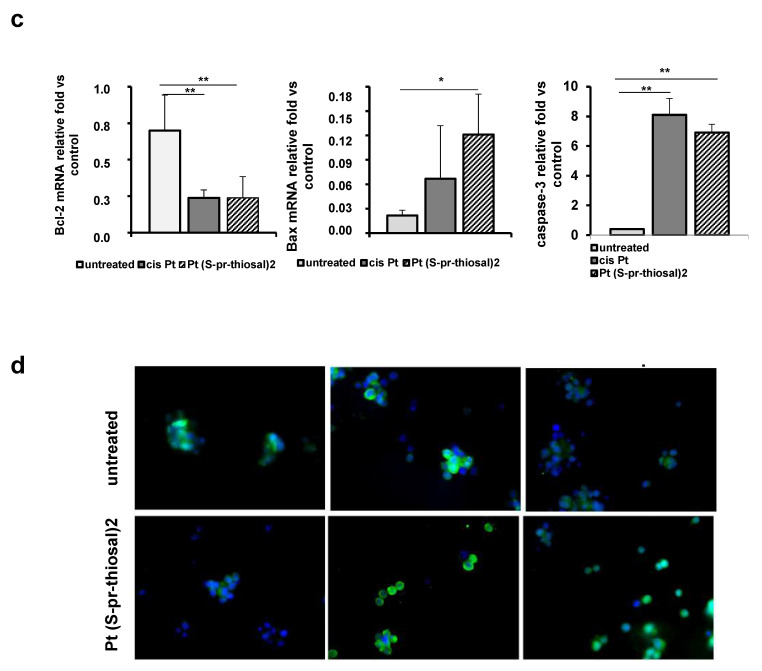
Pt(*S*-pr-thiosal)2 enhances expression of proapoptotic molecules, while reducing expression of antiapoptotic molecules in BCL1 cells. (**a**) The graphs show the mean for the percentage of Noxa and Bcl2 positive BCL1 cells. The statistical significance of the difference in the percentage of apoptotic cells was determined by Student’s *t* test; * *p* < 0.05, ** *p* < 0.01 and indicates the difference in the percentage of BCL1 cells treated with platinum complex at 0.05 mg/mL and all three other groups of BCL1 cells (untreated; treated with cisplatin; and treated with platinum complex at 0.01 mg/mL). (**b**) Representative histograms show the expression of Bcl-2 and Noxa in untreated, cisplatin-treated, and Pt(*S*-pr-thiosal)2 treated BCL1 cells; (**c**) Mean values +SD for expression of mRNA for Bcl2, Bax and caspase-3 detected in BCL1 cells by real-time qRT-PCR for 5 samples per group are shown. The statistical significance was determined by Student’s *t* test; * *p* < 0.05, ** *p* < 0.01. (**d**) Immunofluorescence staining for (green stained proteins Bcl-2 (left), Bax (middle), and active form of caspase 3 (right); DNA stained blue) (DAPI)) in BCL1 cells untreated and treated with platinum complex for 12 h at a dose of 0.05 mg/mL, magnification 200×.

Since Pt(*S*-pr-thiosal)2 complex has previously shown the strongest cytotoxic effect on murine CLL cells, BCL1 [22], the potential of this complex to induce BCL1 cell apoptosis was analyzed. Flow cytometry analysis of Annexin V and propidium iodide-stained BCL1 cells 24 h after treatment with Pt(*S*-pr-thiosal)2, in two different doses of 0.1 mg/mL and 0.05 mg/mL revealed that the tested complex induced apoptosis of BCL1 cells (Figure 1). Twenty-four hours after platinum complex exposure (concentration 0.01 mg/mL), the percentage of BCL1 cells that are in the early stages of apoptosis (AnnexinV + PI cells) is not higher compared to the percentage of early apoptotic untreated and cisplatin-treated BCL1 cells, but the percentage of late apoptotic (AnnexinV + PI +) cells treated with the platinum complex is higher than the percentage of these cells after cisplatin treatment (Figure 1a). However, exposure of BCL1 cells to a higher dose of platinum complex (0.05 mg/mL) significantly increased the percentage of both early, and especially late apoptotic cells compared to cisplatin-treated cells (Figure 1a,b). Further analysis was done in order to explore the effect of Pt(*S*-pr-thiosal)2 treatment on the expression of pro- and anti- apoptotic molecules. The percentage of Noxa-positive BCL1 cells was significantly higher after treatment of these cells with Pt(*S*-pr-thiosal)2 at both test concentrations (0.01 and 0.05 mg/mL) compared to untreated, as well as cisplatin-treated BCL1 cells (Figure 2a). There was no difference in the percentage of BCL1 cells expressing Noxa protein treated with two different concentrations of the tested platinum complex. The percentage of BCL1 cells expressing Bcl-2 was significantly lower after treatment with Pt(*S*-pr-thiosal)2, compared to the percentage of Bcl-2 positive cisplatin-treated BCL1 cells (Figure 2a). Cisplatin treatment did not affect the expression of Noxa and Bcl2 protein in BCL1 cells. Histograms in Figure 2b show that the Pt(*S*-pr-thiosal)2 complex reduces the expression of Bcl-2, but also increases the expression of the proapoptotic protein Noxa in BCL1 cells.

An analysis of the expression of molecules, Bcl2, Bax, and caspase-3, (at the mRNA level), whose relative ratio determines cell fate, apoptotic death, or survival was further performed. The tested platinum complex significantly reduced Bcl2 mRNA expression, similarly to cisplatin (Figure 2c), and significantly increased Bax mRNA expression when compared to untreated cells (Figure 2c). In addition, platinum complex treatment, similarly to cisplatin, significantly increased caspase-3 expression in BCL1 cells (Figure 2c). Decreased Bcl2 and increased Bax and active caspase-3 expression were observed in BCL1 cells by the immunofluorescence staining analysis (Figure 2d). These results suggest that the Pt(*S*-pr-thiosal)2, induces caspase-mediated apoptosis of BCL1 cells.

**Figure 3:**
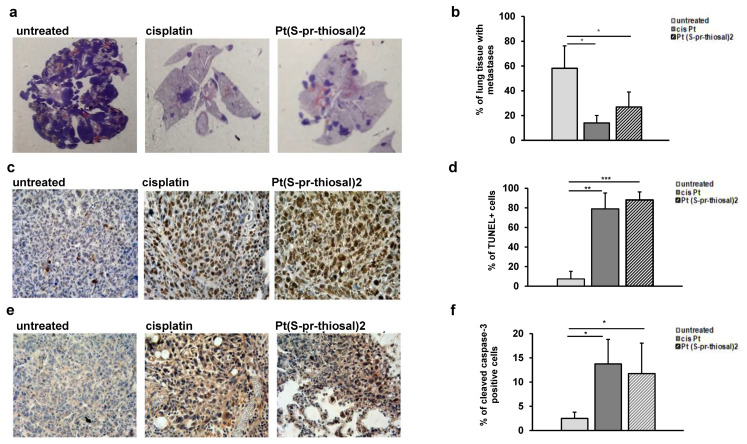
Pt(*S*-pr-thiosal)2 induces significant apoptotic effect in vivo. (**a**) Representative sections of lung tissue obtained from mice injected intravenously with BCL1 cells and treated with platinum complex, cisplatin, or saline, stained with hematoxylin and eosin. (**b**) The mean value ± SD of the percentage of lung tissue affected by metastases (n = 10 for each of the groups) calculated by analyzing lung tissue preparations stained with hematoxylin and eosin in Image J. (**c**) Representative lung sections obtained four weeks after application of BCL1 cells stained by the TUNEL method. (**d**) The percentage of TUNEL-positive nuclei (brown) presented as mean values + SD for 5 mice per group. (**e**) Representative immunohistochemistry of active caspase-3 expression in tumor tissue in the lungs of untreated mice and mice treated with cisplatin and complex. (**f**) The percentage of active caspase-3 positive tumor cells (brown color) in relation to the total number of cells in the visual field presented as mean values + SD. * *p* < 0.05, ** *p* < 0.01, *** *p* < 0.001.

As it was clearly shown that the Pt(*S*-pr-thiosal)2, induces apoptotic death of BCL1 cells in vitro, the effect of this complex was further investigated in vivo, using BALB/c mice intravenously injected with 1 × 10^5^ BCL1 cells. Treatment with Pt(*S*-pr-thiosal)2 (4 mg/kg three times per week in nine doses) significantly reduced the percentage of lung tissue affected by metastases in comparison with the group of untreated mice (Figure 3a). The percentage of lung tissue affected by metastases in the group of mice treated with the platinum complex was slightly higher compared to the group of mice treated with cisplatin, but this difference was not statistically significant (Figure 3b). In order to show that Pt(*S*-pt-thiosal)2 induces apoptosis of BCL1 cells also in vivo and thus reduces the presence of lung metastases, fragmented DNA, a marker of late apoptosis was analyzed in lung tissue sections by TUNEL assay. Figure 3c clearly shows the presence of TUNEL-positive cells in the tumor tissue in lungs of mice treated with cisplatin and the Pt(*S*-pt-thiosal)2 complex. The intensity of the brown color (TUNEL positive cells) is higher in the lung sections of mice treated with platinum complex compared to the group of mice treated with cisplatin. Quantitative analysis showed that the percentage of TUNEL-positive cells was significantly higher in the group of mice treated with the Pt(*S*-pt-thiosal)2 complex compared to the group of untreated mice (Figure 3d). Treatment of mice with cisplatin has a similar effect (Figure 3c,d).

Immunohistochemical analysis of the expression of active caspase-3 in the lung tissue of treated and untreated mice showed that apoptosis pf tumor cells induced in vivo by the Pt(*S*-pr-thiosal)2 complex are most probably caspase-dependent. The expression of active caspase-3 is higher in lung sections of mice treated with both cisplatin and Pt(*S*-pr-thiosal)2 compared to sections obtained from untreated mice (Figure 3e). Quantitative analysis of immunohistochemically stained lung sections showed that the percentage of tumor cells expressing the active form of caspase-3 was significantly higher in the group of mice treated with Pt(*S*-pr-thiosal)2 complex compared with the group of untreated mice (Figure 3f). There was no difference in the percentage of caspase-3 positive cells between the cisplatin-treated groups of mice and the Pt(*S*-pr-thiosal)2 complex (Figure 3f).

### 2.2. The Pt(S-pr-thiosal)2 Complex Causes BCL1 Cell Arrest in the G0/G1 Phase of Cell Cycle and Has an Antiproliferative Effect

**Figure 4:**
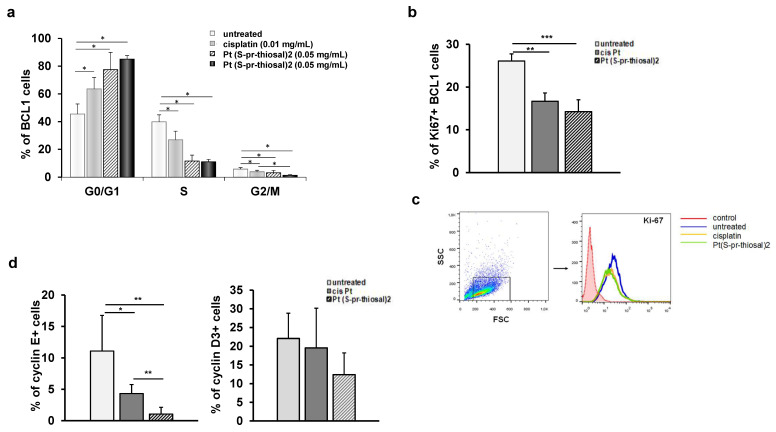
Pt(*S*-pr-thiosal)2 has antiproliferative effect on BCL1 cells. (**a**) The graph shows the mean percentage of BCL1 cells by phases of the cell cycle + SD; (**b**) Percentage of Ki67+ BCL1 cells + SD; (**c**) Representative histograms show Ki-67 expression in untreated, cisplatin-treated and Pt(*S*-pr-thiosal)2 treated BCL1 cells. (**d**) Percentage of cyclin E+ and cyclin D3+ BCL1 cells + SD from three independent experiments, for 3 replicates. Statistical significance was determined by Student’s *t* test, * *p* < 0.05, ** *p* < 0.01, *** *p* < 0.001. (**d**) representative histograms show Ki-67 expression in untreated, cisplatin-treated and Pt(*S*-pr-thiosal)2 treated BCL1 cells.

To examine whether the Pt(*S*-pr-thiosal)2 complex affects the cell cycle in BCL1 cells and thus contributes to cell death, BCL1 cells were treated with the platinum complex in two different doses for 12 h and then the distribution cells by phases of the cell cycle was analyzed by flow cytometry. The highest percentage of BCL1 cells treated with Pt(*S*-pr-thiosal)2 complex (both tested doses, 0.01 mg/mL and 0.05 mg/mL), similar to BCL1 cells treated with cisplatin, are in the G0/G1 phase of the cell cycle (Figure 4a) and this percentage is significantly higher compared to the percentage of untreated BCL1 cells in the G0/G1 phase of the cell cycle. On the other hand, treatment with the Pt(*S*-pr-thiosal)2 complex significantly reduced the percentage of BCL1 cells in the S phase, indicating that the complex realizes an antiproliferative effect (Figure 4a). Moreover, the percentage of BCL1 cells treated with Pt(*S*-pr-thiosal)2 complex that are in the S phase of the cell cycle is significantly lower compared to the percentage of BCL1 that are in this phase after cisplatin treatment (Figure 4a). The Pt(*S*-pr-thiosal)2 complex, as well as cisplatin, also significantly reduced the percentage of BCL1 cells in the G2/M phase of the cell cycle. There is no difference in the effect of two different doses of the Pt(*S*-pr-thiosal)2 complex on the distribution of BCL1 cells by cell cycle phases.

The finding of a significant decrease in the percentage of BCL1 cells expressing the Ki67 proliferation marker after the treatment with the Pt(*S*-pr-thiosal)2 complex (Figure 4b) indicates the antiproliferative effect of the examined complex and is consistent with the previous finding of a significantly reduced percentage of BCL1 cells in the S phase of the cell cycle (Figure 4a).

A significant decrease in Ki-67 expression in BCL1 cells was observed after treatment with Pt(*S*-pr-thiosal)2 complex compared to untreated but also cisplatin-treated BCL1 cells (Figure 4c).

The cyclins and cyclin-dependent kinases control the progression of cells through the cell cycle by regulating the transition from one phase to another [26,27]. Cyclins positively regulate cyclin-dependent kinases, and cyclin-dependent kinase inhibitors play a role in negative cell cycle regulation [28]. As cyclins D and E are marked as cyclins of the G1 phase of the cell cycle [29,30], the percentage of BCL1 cells that express cyclin E and cyclin D3 after treatment with Pt(*S*-pr-thiosal)2 complex was further analyzed by flow cytometry. As seen in Figure 4d, treatment with Pt(*S*-pr-thiosal)2 complexes significantly reduced the percentage of BCL1 cells expressing cyclin E. Treatment of BCL1 cells with cisplatin also significantly reduced the percentage of cells that express cyclin E (Figure 4d). However, the percentage of cyclin E positive BCL1 cells was significantly lower in the group of cells treated with Pt(*S*-pr-thiosal)2 complex compared to cisplatin-treated cells (Figure 4d).

Treatment with the Pt(*S*-pr-thiosal)2 complex also reduced the percentage of BCL1 cells expressing cyclin D3, although this difference did not reach statistical significance (Figure 4d).

**Figure 5:**
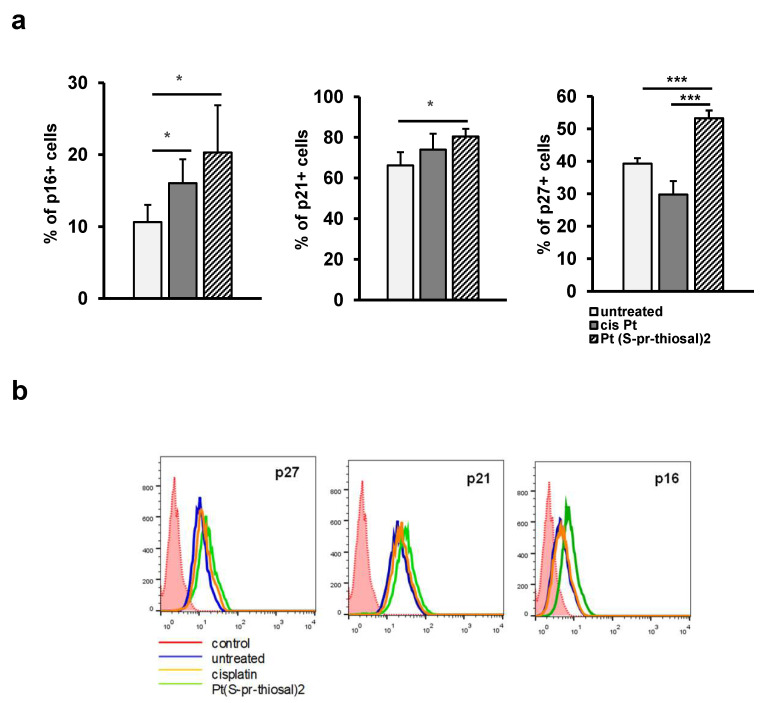
Pt(*S*-pr-thiosal)2 increases the percentage of BCL1 cells expressing cyclin-dependent kinase inhibitors p16, p21, and p27. After treatment with Pt(*S*-pr-thiosal)2 complex (dose 0.05 mg/mL) and cisplatin for 12 h, BCL1 cells were stained with anti-p16, anti-p21, and anti-p27 antibodies, and the percentage of positive cells was detected by flow cytometry. (**a**) The graph shows the mean values of the percentage of positive BCL1 cells +SD for 3 replicates. Statistical significance was determined by Student’s *t* test; * *p* < 0.05; *** *p* < 0.001. (**b**) Representative histograms show the expression of p27, p21, and p16 in untreated, cisplatin-treated, and Pt(*S*-pr-thiosal)2 treated BCL1 cells.

The expression of negative cell cycle regulators, p16, p21, and p27, cyclin-dependent kinase inhibitors that control the transition from G1 to S phase of the cell cycle was also analyzed in treated and untreated cells. A significantly higher percentage of BCL1 cells express cyclin/cyclin-dependent kinase complex inhibitors p16, p21, and p27 after treatment with Pt(*S*-pr-thiosal)2 (0.05 mg/mL) complex compared to untreated cells (Figure 5a). The percentage of BCL1 cells expressing p27 was significantly higher in the group of cells treated with the Pt(*S*-pr-thiosal)2 (0.05 mg/mL) complex compared to cisplatin-treated cells (Figure 5a). Higher p16 expression was also observed in BCL1 cells treated with the Pt(*S*-pr-thiosal)2 complex compared to cisplatin-treated and untreated BCL1 cells (Figure 5b).

### 2.3. The Pt(S-pr-thiosal)2 Complex Attenuates the Expression of Phosphorylated STAT3 and Molecules Whose Expression Is Regulated by STAT3 in BCL1 Cells

**Figure 6:**
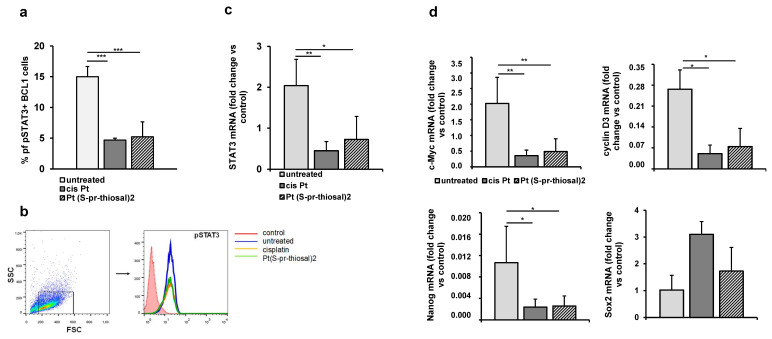
Pt(*S*-pr-thiosal)2 decreases the expression of phosphorylated STAT3 and STAT3 regulated genes in BCL1 cells. (**a**) The percentage of (phospho Y705) STAT3 positive BCL1 cells was detected by flow cytometry, the relative expression of mRNA for (**c**) STAT3 and (**d**) c-Myc, cyclin D3, Nanog, and Sox2, normalized to β-actin, was analyzed by real-time qRT-PCR and presented as mean values + SD for 5 samples per group. The statistical significance was determined by Student’s *t* test; * *p* < 0.05, ** *p* < 0.01, *** *p* < 0.001. (**b**) Representative histograms show the expression of phosphorylated STAT3 in untreated, cisplatin-treated, and Pt(*S*-pr-thiosal)2 treated BCL1 cells.

Overexpression of the STAT3 molecule has been shown in CLL cells and it has also been shown that it is constitutively phosphorylated at (Tyr) 705 and (Ser) 727 positions, while in normal peripheral B lymphocytes or CD5+ B cells isolated from tonsils, STAT3 is unphosphorylated [31,32]. Since STAT3 plays one of the key roles in cancer development and progression and mediates the interaction of CLL and bone marrow cells which is crucial for maintaining viable CLL cell populations even after chemotherapy [24], the effect of Pt(*S*-pr-thiosal)2 on the level of phosphorylated STAT3 in BCL1 cells was analyzed by flow cytometry. The percentage of BCL1 cells expressing phosphorylated STAT3 (phospho Y705) after treatment with Pt(*S*-pr-thiosal)2 complex was significantly lower compared to the untreated BCL1 cells (Figure 6a). Expression of STAT3 at the level of mRNA was also statistically significantly lower in BCL1 cells treated with the Pt(*S*-pr-thiosal) 2 complex than in untreated cells (Figure 6c).

The expression of phosphorylated STAT3 in cisplatin-treated BCL1 cells and the Pt(*S*-pr-thiosal)2 complex compared to untreated cells (Figure 6b) detected by flow cytometry was markedly reduced.

Further analysis showed that the Pt(*S*-pr-thiosal)2 complex significantly reduced the expression of STAT3-regulated molecules, cyclin D3, and c-Myc (Figure 6d), protooncogenes that control progression through the cell cycle [33]. Moreover, treatment of BCL1 cells with the Pt(*S*-pr-thiosal)2 complex also significantly reduced the expression of Nanog molecule (Figure 6d) expressed in stem cells of solid tumors and leukemias [34]. Cisplatin, similarly to the examined complex, significantly reduced the presence of phosphorylated STAT3, as well as mRNA expression for STAT3 (Figure 6a–c), cyclin D3, c-Myc, and Nanog (Figure 6d). Treatment with Pt(*S*-pr-thiosal)2 complex as well as cisplatin did not affect the expression of Sox2 at the level of mRNA (Figure 6d).

### 2.4. Pt(S-pr-thiosal)2 Complex Attenuates Expression of Phosphorylated NF-κB in BCL1 Cells

**Figure 7:**
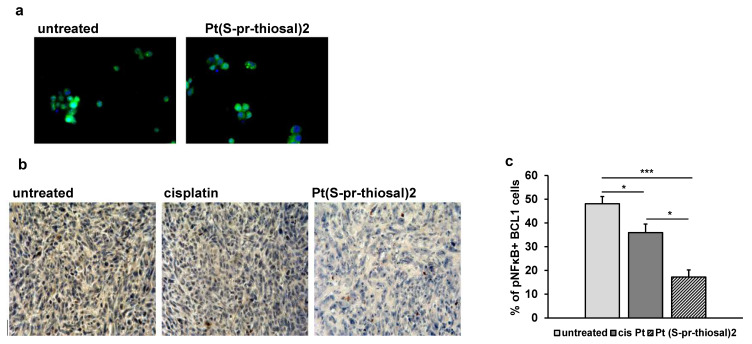
The expression of the phosphorylated form of NF-κB is decreased in BCL1 cells after exposure to Pt(*S*-pr-thiosal)2. (**a**) Immunofluorescence (green stained phosphorylated NF-κB and DNA stained blue) (DAPI)) BCL1 cells untreated and treated with platinum complex for 12 h, magnification 200×. (**b**) Representative images of phosphorylated NF-κB expression in tumor tissue (detected by immunohistochemistry) in the lungs of untreated mice and mice treated with cisplatin and Pt(*S*-pr-thiosal)2, four weeks after intravenous administration of BCL1 cells. (**c**) The percentage of positive cells (expressing NF-κB) in relation to the total number of tumor cells in the field of view presented as the mean values + SD for 5 mice per group. Statistical significance was determined by Student’s *t* test; * *p* < 0.05; *** *p* < 0.001.

Neoplastic B lymphocytes isolated from the peripheral blood of persons with chronic lymphocytic leukemia always show increased NF-κB signaling activity compared to circulating normal B lymphocytes [35,36,37]. Since NF-κB activation is associated with stimulation of CLL cell survival, it can be a good target for potential new drugs, hence the effect of Pt(*S*-pr-thiosal)2 complex on the expression of the active form of NF-κB in BCL1 cells was analyzed. In vitro treatment of BCL1 cells with the Pt(*S*-pr-thiosal)2 complex reduces the expression of the phosphorylated form of NF-κB (Figure 7a). It has been shown that the microenvironment in the lymph nodes in chronic lymphocytic leukemia promotes BCR signals and thus enhances NF-κB signaling pathway activation [38], most likely to maintain CLL cell proliferation and survival in vivo. The effect of Pt(*S*-pr-thiosal)2 complexes on phosphorylated NF-κB expression in vivo in lung metastases four weeks after tumor cell administration was analyzed. As seen in Figure 7b, the Pt(*S*-pr-thiosal)2 complex reduces the presence of phosphorylated NF-κB in tumor tissue in the lung. Moreover, only a few tumor cells containing the phosphorylated form of NF-κB in the lungs of mice treated with the Pt(*S*-pr-thiosal)2 complex, in contrast to the tissues of cisplatin-treated and untreated mice where half of the tumor cells were positive for phosphorylated NF-κB (Figure 7b). Quantitative analysis of lung sections showed that the percentage of tumor cells expressing the phosphorylated form of NF-κB was significantly lower in the group of mice treated with the Pt(*S*-pr-thiosal)2 complex compared to the group of untreated mice (Figure 7c). Similarly, the percentage of tumor cells expressing phosphorylated NF-κB in the group of mice treated with cisplatin was significantly lower compared to the group of untreated mice, but this percentage was also significantly higher compared to the group of mice treated with Pt(*S*-pr-thiosal)2 (Figure 7c).

## 3. Discussion

In this study, in vitro antitumor activity of Pt(IV) complex with thiosalicylic acid derivative toward murine B cell leukemia cells, BCL1, the potential mechanism of cytotoxic activity of this complex and the ability to reduce the growth of leukemia cells in vivo were investigated. The findings from the in vitro cytotoxicity study we previously reported [20] and from this study (Figure 2), are consistent with the findings of in vivo experiments (Figure 3). Pt(*S*-pr-thiosal)2 complex administered intraperitoneally from the seventh day after intravenous administration of BCL1 cells in a total of nine doses, reduces the number and size of lung metastases (Figure 3a) and significantly reduces the percentage of lung tissue affected by metastases (Figure 3b). The previous study has shown that BCL1 cells after intravenous administration in the final stages of disease infiltrate lymph nodes and lungs where they form metastases [38], resembling those on Richter transformation of CLL in humans and development of large B-cell lymphoma or Hodgkin’s variant of Richter transformation [12]. Similar to the results of our study, a significant therapeutic effect of the platinum(IV) complex was shown in vivo in the L1210 model of leukemia [39]. Oxaliplatin-based platinum(IV) complexes significantly increased the survival of mice with leukemia [40].

The results of numerous studies indicate that different platinum complexes induce apoptotic death of tumor cells [41,42,43,44,45,46], so the ability of Pt(*S*-pr-thiosal)2 complexes to induce BCL1 cell apoptosis was analyzed. Pt(*S*-pr-thiosal)2 complex at both administered doses induced apoptosis of BCL1 cells (Figure 1). The percentage of early and late apoptotic BCL1 cells after lower dose treatment was similar to the percentage of these cells after cisplatin treatment (0.01 mg/mL), but treatment with a higher dose of complex significantly increased this percentage (Figure 1a). Chemotherapeutics most commonly activate the mitochondrial pathway of apoptosis [44,47,48] when the permeability of the outer mitochondrial membrane increases [49] and proapoptotic caspase-activating molecules are released from mitochondria resulting in apoptotic DNA fragmentation and cell death [50,51,52,53]. Members of the BCL2 (from B cell lymphoma 2) family of proteins are major regulators of cell survival and death [54]. This family contains three functionally different subgroups: antiapoptotic proteins (BCL2, BCL-XL, BCL-W, MCL1, and A1/BFL-1), proapoptotic effector proteins (BAX and BAK), and proapoptotic BH3-only proteins (BIM, PUMA, BID, BAD, BIK, BMF, NOXA and HRK) [55,56]. Molecules that mimic the BH3 domain show a significant effect in tumor therapy [57]. Our study showed a significant increase in the percentage of BCL1 cells expressing the pro-apoptotic molecule Noxa after treatment with the platinum complex at both tested doses compared to cisplatin (Figure 2a). Noxa is a BH3-only pro-apoptotic protein that inactivates the anti-apoptotic molecule Mcl1 and thus plays a key role in inducing tumor cell death by cisplatin [58,59]. Pharmacologically induced Noxa protein expression in lymphoma cells has been shown to reduce Mcl1 protein expression and thus increase the vulnerability of lymphoma cells in vitro and in vivo [60]. Overexpression of anti-apoptotic molecule Bcl-2 is a hallmark of CLL and B cell lymphomas [61]. Here we have found a significantly lower percentage of BCL1 cells expressing Bcl-2 (Figure 2a) after treatment with the Pt(*S*-pr-thiosal)2 complex compared to cisplatin, indicating a potentially stronger apoptotic effect of the new platinum complex toward BCL1 cells compared to cisplatin. Further, a significant decrease in Bcl-2 expression was associated with a significant increase in caspase-3 expression in BCL1 cells treated with both cisplatin and Pt(*S*-pr-thiosal)2 complex compared to untreated cells (Figure 2c,d). The Pt(*S*-pr-thiosal)2 complex also significantly increased the expression of Bax molecules in BCL1 cells (Figure 2c). Dysregulation of apoptosis in malignant B cells is due to overexpression of Bcl2 protein and very often disturbing relationship between Bcl2 and Bax protein [61,62]. The finding of a significant increase in Bax expression and a decrease in Bcl2 expression in BCL1 cells after treatment with Pt(*S*-pr-thiosal)2 indicates a significant potential of this complex to induce apoptotic death of leukemia cells. In line with this is a significant increase in caspase-3 expression in treated cells, effector molecules that play a central role in the process of chromatin condensation and DNA fragmentation which are the main features of the cell in apoptosis [63].

Mutation of TP53, a major player in DNA damage response, is one of the few genetic lesions present in 5–7% of all CLL cases [64], and also aggressive B cell lymphoma [65] TP53 activates the intrinsic apoptotic pathway by inducing several BH3- only proteins [66], while TP53 disruption is recognized as a strong predictor of chemoimmunotherapy resistance in patients with B cell lymphoma [67]. It has been shown that agents that directly antagonize BCL2 function such as venetoclax may restore apoptosis in cells with disrupted TP53 and thus may overcome the negative prognostic impact of TP53 disruption [68]. In line with this, and having in mind attenuation of Bcl-2 by Pt(*S*-pr-thiosal)2 complex in BCL1 cells and increased percentage of apoptotic BCL1 cells, it could be assumed that the newly synthesized Pt(*S*-pr-thiosal)2 complex could achieve antitumor (proapoptotic effect) in chemotherapy-resistant B cell malignancies.

The ability of the Pt(*S*-pr-thiosal)2 complex to induce apoptosis of tumor cells in vivo has been demonstrated in metastases in lung tissue. It has been clearly shown that the Pt(*S*-pr-thiosal)2 complex significantly increases the percentage of TUNEL-positive tumor cells in lung tissue (Figure 3c,d). The TUNEL method detects apoptotic cells during the process of intensive DNA degradation that is characteristic of the late phase of apoptosis [69]. Associated with this finding is an increase in the percentage of tumor cells in lung metastases in which the active form of caspase-3 is detected (Figure 3e,f). These findings indicate that the Pt(*S*-pr-thiosal)2 complex induces, most probably, caspase-dependent apoptosis of BCL1 cells.

Disturbed regulation of genes encoding molecules involved in cell cycle regulation contributes to malignant clone expansion [70], so the examination of new potential drugs that would target so-called cell cycle checkpoints is actual. In line with the findings of lower BCL1 cell viability and higher expression of apoptotic proteins associated with higher apoptosis of BCL1 cells after treatment with Pt(*S*-pr-thiosal)2, a significantly lower percentage of BCL1 cells expressing cyclin E and lower mRNA expression of cyclin D3 was found after treatment with this complex, compared to both untreated and cisplatin-treated BCL1 cells (Figure 4d), as well as a lower percentage of cells expressing the Ki67 proliferation marker (Figure 4b). Higher expression of cyclin D3 in various tumors including lymphoma is an unfavorable prognostic biomarker that affects the outcome of chemotherapy makers [71]. Cyclin D3 interacts with cyclin-dependent kinase 4/6, cdk4/6, and this interaction is crucial for cell progression through the G1 phase of the cell cycle in human B lymphocytes and is blocked by the cdk inhibitor p27 [72]. Cyclin E is overexpressed in a number of tumors including leukemias [73,74,75]. Cyclin E is maximally expressed during the transition period from the G1 to S phase of the cell cycle, and its activity is limited by the cdk inhibitor p27 [76]. In accordance with the above functions of cyclin E and cyclin D3 and data on the reduced expression of these two cyclins in BCL1 cells treated with Pt(*S*-pr-thiosal)2 is the finding of an increased percentage of BCL1 cells treated with complex in the G1 phase of the cell cycle (Figure 4a). It can be concluded that the Pt(*S*-pr-thiosal)2 complex stops the expression of two cyclins that play a key role in the transition of cells from the G1 to S phase of the cell cycle leading to the accumulation of BCL1 cells in the G1 phase, which is closely related to the reduction of BCL1 cells in the S phase of the cell cycle. Pt(*S*-pr-thiosal)2 has an antiproliferative effect, which is indicated by a decrease in the percentage of Ki-67 positive BCL1 cells (Figure 4b) after treatment. Malignant B cells in various lymphomas and CLL are known to express Ki-67 [77,78].

Stopping of BCL1 cells in the G1 phase after treatment with Pt(*S*-pr-thiosal) 2 complex was associated with a significant increase in the percentage of BCL1 cells expressing cyclin-dependent kinase inhibitors p16, p21, and p27 (Figure 5a). The inhibitor of a cyclin-dependent kinase, p27, binds to both cyclin D-cdk4 and cyclin E-cdk2 complex [79], inhibits them and plays a key role in the regulation of cell cycle progression in lymphocytes [80,81]. Altered expression of this protein plays a role in the pathogenesis of lymphoproliferative diseases [82]. The expression of p21 and p16 is decreased in cells exposed to various stimuli that promote malignant transformation [83,84], and the ability of both of these molecules to induce irreversible cell cycle arrest indicates the role of both molecules in preventing tumor development [85,86,87]. The tumor suppressive role of p16 and p21 molecules confirms the development of tumors in mice with deletion of genes for these two molecules very early in life [88,89,90,91] and illustrates their importance in suppressing tumor development in vivo. Otherwise, p16, by interfering with the function of the cyclin D cdk4/6 complex that results in a lack of Rb phosphorylation [92], slows the progression of the cell cycle from G1 to the S phase, stops the cells in the G1 phase, and blocks cell division. Similarly, p21 inhibits the activity of the cyclin D cdk4/6 complex and cyclin E cdk2 complex and thus blocks cell progression from G1 to the S phase of the cell cycle [93,94].

Interruption of cell progression from the G1 to S phase of the cell cycle accelerates apoptosis [95], so our results indicate that the Pt(*S*-pr-thiosal)2 complex by increasing expression of cyclin-dependent kinase inhibitors p16, p21, and p27 and by decreasing cyclin E and D3 expression, blocks cell cycle progression and proliferation of BCL1 cells which likely results in BCL1 apoptosis.

Constitutive expression of STAT3 molecules has been confirmed in a variety of leukemias [31,96,97,98,99] including CLL and B cell lymphomas [100,101]. STAT3 is the most commonly activated transcription factor in tumors where it regulates processes characteristic of tumor cells such as uncontrolled proliferation, resistance to apoptosis, angiogenesis, and immune response escape [102,103]. A lower percentage of BCL1 cells expressing the active form of STAT3 (phosphorylated STAT3) and significantly lower STAT3 mRNA expression in BCL1 cells were detected after treatment with the Pt(*S*-pr-thiosal)2 (Figure 6a–c).

Phosphorylated STAT3 dimerizes and goes to the nucleus where it binds to promoters of target gene sequences, BCL2, Pim1, BCL-XL, cyclin D1, p21, c-MYC, Sox2, Nanog, and cyclin D3 [104]. Consistent with the above, our results show significantly lower mRNA expression for c-Myc, cyclin D3, and Nanog in BCL1 cells treated with Pt(*S*-pr-thiosal)2 complex compared to untreated cells (Figure 6d) and lower Bcl2 expression (Figure 2). STAT3 also, by inducing the expression of cyclin D1, D2, D3, and cyclin-dependent kinase Cdc25A and by the associated reduction in expression of cyclin-dependent kinase inhibitors p21 and p27, plays a key role in cell cycle progression from the G1 to the S phase [105,106]. Our results suggest that Pt(*S*-pr-thiosal)2 complex, by disrupting the expression of the active form of STAT3, disrupts the ratio of Bcl2 and Bax, indirectly reducing the expression of c-Myc, a transcription factor that regulates the expression of genes involved in proliferation [107], cyclin D3, and increases the expression of inhibitors of cyclin-dependent kinase p21 and p27 resulting in the arrest of BCL1 cells in the G1 phase of the cell cycle, inhibition of proliferation and induction of apoptosis.

Nanog, Sox2, and c-Myc are transcription factors expressed in embryonic stem cells [108,109], but the aberrant expression of these molecules has been observed in various tumors and is associated with the development of cancer stem cell phenotypes [110,111]. In this study, significantly lower Nanog and c-Myc expression were shown in cells treated with Pt(*S*-pr-thiosal)2 compared to untreated cells (Figure 6). The correlation between the reduced percentage of Y705 phosphorylated STAT3 in BCL1 cells treated with Pt(*S*-pr-thiosal)2 complex and the reduced gene expression for Nanog, c-Myc and cyclin D3 indicates that this complex by modulating the STAT3 signaling affects the BCL1 cell phenotype and reduces cancer stemness.

Also, the STAT3 molecule interacts directly with NF-κB and in the form of heterodimers goes to the nucleus where it affects the expression of the molecules which stimulates the proliferation of cancer cells [112]. In addition, the active form of phosphorylated NF-κB is constitutively present in malignant B cells and affects both the development and progression of the disease by modulating the expression of target genes [7,8]. We have found weaker expression of phosphorylated NF-κB in BCL1 cells in vitro treated with Pt(*S*-pr-thiosal)2 complex compared to untreated cells (Figure 7a), while in the tumor tissue of mice treated with this complex, a significantly lower percentage of tumor cells which express the active form of NF-κB was observed (Figure 7b,c). These findings indicate that the Pt(*S*-pr-thiosal)2 complex can at least partially achieve its antitumor activity by modulating the activation of the NF-κB signaling pathway.

## 4. Materials and Methods

### 4.1. Tumor Cell Line

BCL1 (mouse B cell leukemia lymphoma), cells were purchased from the American Type Culture Collection (TIB-197; ATCC, Manassas, VA, USA). The cells were maintained in RPMI 1640 (Sigma-Aldrich, St. Louis, MO, USA) supplemented with 15% fetal bovine serum (FBS, Sigma-Aldrich), 0.05 mM 2-mercaptoethanol (Sigma-Aldrich) penicillin (100 IU/mL), and streptomycin (100 μg/mL) in a humidified atmosphere of 95% air/5% CO_2_ at 37 °C. Only cell suspensions with >95% viable cells were used in all in vitro and in vivo experiments. Viability of tumor cells was determined using trypan blue.

### 4.2. Animals

Experimental animals, 8–10-week-old male BALB/C mice were used and housed in a temperature-controlled environment with a 12 h light-dark cycle, fed *ad libitum* and observed daily. Also, the mice were equalized in weight and randomized into experimental or control groups (n = 10 animals per group). All experiments were approved by and conducted in accordance with the Guidelines of the Animal Ethics Committee of the Faculty of Medical Sciences of the University of Kragujevac, Serbia.

### 4.3. Experimental Model of BCL1 Leukemia and Platinum Complex Treatment

Mice were administered intravenously 10^5^ BCL-1 cells into the lateral tail vein. From the seventh day after tumor cell administration, mice received Pt(*S*-pr-thiosal)2) and cisplatin, intravenously 3 times a week (9 doses in total). The dose of the complex used for in vivo assays was determined based on the IC50 values obtained in previous study (11). Mice were treated with Pt(*S*-pr-thiosal)2 complex at a dose of 4 mg/kg, cisplatin at the same dose was used as a control substance, and the third group of mice received saline intraperitoneally. As the BCL1 line often metastasizes to the lungs, the lungs were extirpated and routinely fixed in formalin after mouse sacrifice.

### 4.4. Quantification of Lung Metastases

For pathohistological verification of lung metastases, at least 5 sections of tissue stained with hematoxylin and eosin were taken from each mouse by standard procedure. Sections were imaged using a light microscope (BX51, Olympus, Tokyo, Japan). The percentage of lung tissue affected by metastases was then semiquantitatively determined using ImageJ software.

### 4.5. In Situ TUNEL Staining

In order to assess apoptotic cells within lung metastases sections, the TUNEL reaction was performed. Formalin-fixed, paraffin-embedded tissue sections were stained with In Situ *Cell Death Detection Kit*, POD (Merck, Kenilworth, NJ, USA) following the protocol of the manufacturer. To yield the characteristic brown color for the nuclei, DAB (3,3′-diaminobenzidine) was used. Following rinsing, the slides were counterstained with hematoxylin solution. Photomicrography was performed with a digital camera mounted on the light microscope. The negative control was performed by omitting TdT reaction step. For quantification of the TUNEL-positive (brown) nuclei 400× magnification was used in five random fields representing at least 1000 neoplastic nuclei. The data were summarized as the mean percentage of positive cells (four tumors per group)

### 4.6. Assessment of Cell Death by Flow Cytometry

For the detection of apoptosis, the Annexin V binding capacity of treated cells was examined by flow cytometry using Annexin V Fluorescein isothiocyanate (FITC). BCL1 cells were incubated with the complex at concentrations 0.01 and 0.05 mg/mL, cisplatin 0.01 mg/mL, or with media alone (control) for 24 h at 37 °C in an atmosphere containing 5% CO_2_ and at absolute humidity. Following the incubation, all cells were washed in PBS, centrifuged and resuspended in 100 μL of ice-cold binding buffer [10 × binding buffer: 0.1 M Hepes/NaOH (pH 7.4), 1.4 M NaCl, 25 mM CaCl_2_] at the concentration of 1 × 10^6^/mL. Annexin V-FITC (BD Pharmingen) and PI (Sigma Aldrich) were added to 100 μL of cell suspension and incubated for 15 min at room temperature (25 °C) in the dark. After the incubation, 400 μL of 1× binding buffer was added to each tube, and stained cells were analyzed within 1 h using Fluorescence-activated cell sorting (FACS) Calibur Flow Cytometer (BD Biosciences, San Jose, CA, USA). The data were analyzed using FlowJo Software (Tree Star, Ashland, OR, USA). Measurements were presented as the density plots of Annexin V-FITC and PI stainings.

### 4.7. Cell Cycle Analysis

After 12 h treatment with platinum complex at concentrations 0.01 and 0.05 mg/mL, BCL1 cells were stained with Vybrant^®^ DyeCycle™ Ruby stain according to the manufacturer’s instructions. Cell cycle distribution was analyzed by FACS Calibur flow cytometer (BD Biosciences, San Jose, CA, USA) and the data were processed using FlowJo.

### 4.8. Flow Cytometric Analysis

BCL1 cells, grown in culture plates, were treated with or without Pt(*S*-pr-thiosal)2 (0.05 mg/mL) for 12 h. The treated cells were fixed and permeabilized with permeabilization buffer (BD Bioscience) and incubated with antibodies specific for Bcl-2 (11-6992-42, Thermo Fisher Scientific, Cambridge, UK), Noxa (ab13654, Abcam, Cambridge, UK), STAT3 (IC1799G, Novus Biologicals, San Diego, CA, USA), p16 (ab211542, Abcam, Cambridge, UK), p21 (ab188224, Abcam) and p27 (ab215434, Abcam), cyclin D3 (ab28283, Abcam Cambridge, UK), cyclin E (MA5-14336, Thermo fisher scientifics, Waltham, MA, USA), Ki-67 (151212, BioLegend, San Diego, CA, USA), Noxa (ab13654, Abcam) and Mcl-1 (ab32087, Abcam). For staining p16, p21, and p27 cells were additionally incubated with secondary goat anti-mouse IgG FITC (ab6785, Abcam) or donkey anti-rabbit IgG (ab150073, Abcam). Flow cytometry was performed on FACSCalibur flow cytometer (BD Biosciences). The data were analyzed using FlowJo (Tree Star).

### 4.9. Immunofluorescence Staining

BCL1 cells were seeded in a six-well plate and exposed to the Pt(*S*-pr-thiosal)2 (0.05 mg/mL) for 12 h. Cells were washed twice with phosphate-buffered saline and then fixed in 4% paraformaldehyde at 25 °C for 20 min. For cell staining, rabbit polyclonal antibody specific for Bcl-2 (sc-783, Santa Cruz Biotech Inc., Santa Cruz, CA, USA), Bax (sc-493, Santa Cruz Biotech Inc.), and phospho NF-κB (ab131109 Abcam, Cambridge, UK) and active/cleaved caspase 3 (NB100-56113, Novus Biologicals) were used. After incubation, the cells were washed and treated with appropriate secondary antibody, goat anti-rabbit IgG FITC (Ab6717-1, Abcam). The sections were mounted with ProLong Gold antifade reagent with 4′,6-diamidino-2-phenylindole (DAPI; Invitrogen, Carlsbad, CA, USA). For the cell analysis, fluorescent microscope (Olympus BX 51) was used at 200× magnification.

### 4.10. Immunohistochemistry of Mouse Lung Tissue Samples

Deparaffinized paraffin sections of lung tissue were incubated with primary rabbit antibodies specific for the active form of caspase-3 (NB100-56113, Novus Biologicals) and phosphorylated NF-κB (ab131109 Abcam, Cambridge, UK). Histological sections were visualized using a rabbit detection kit (Expose Rb-Specific HRP/DAB Detection IHC Kit; Abcam) antibodies. The sections were photographed with a digital camera on a light microscope (Olympus BX51). Ten fields per cross-section (magnification 400×) were analyzed and the average number of positive tumor cells relative to the total number of tumor cells per cross-section was detected.

### 4.11. Real-Time PCR Analysis

After 12 h of the treatment with Pt(*S*-pr-thiosal)2 (0.05 mg/mL), total RNA from BCL11 cell was isolated with TRIzol (Invitrogen). The first-strand cDNA was synthesized by high capacity cDNA reverse transcription kit (Applied Biosystems, Foster City, CA, USA). Power SYBR MasterMix (Applied Biosystems) was used for performing quantitative real-time polymerase chain reaction (qRT-PCR) and mRNA-specific primers for *Bax* (forward 5′-ACACCTGAGCTGACCTTG-3′ and reverse 5′-AGCCCATGATGGTTCTGATC-3′), *Bcl-2* (forward 5′-GTGGTGGAGGAACTCTTCAG-3′ and reverse 5′-GTTCCACAAAGGCATCCCAG-3′), *caspase3* (forward 5′-AAATTCAAGGGACGGGTCAT-3′ and reverse 5′-ATTGACACAATACACGGGATCTGT-3′), *STAT3* (forward 5′-GCACCTTGGATTGAGAGTCA-3′ and reverse 5′- CCCAAGAGATTATGAAACACCA-3′), *cyclin D3* (forward 5′- CCGTGATTGCGCACGACTTC-3′ and reverse 5′-TCTGTGGGAGTGCTGGTCTG-3′), *c-Myc* (forward 5′-CGGACACACAACGTCTTGGAA-3′ and reverse 5′-AGGATGTAGGCGGTGGCTTT-3′), *Sox2* (forward 5′-AAAGGGTTCTTGCTGGGTTT-3′ and reverse 5′-AGACCACGAAAACGGTCTTG-3′), *Nanog* (forward 5′-AAGCAGAAGATGCGGACTGT-3′ and reverse 5′-GTGCTGAGCCCTTCTGAATC-3′) and *β-actin* (forward 5′-AGCTGCGTTTTACACCCTTT-3′ and reverse 5′-AAGCCATGCCAATGTTGTCT-3′) as a housekeeping gene. qRT-PCR reactions were initiated with 10-min incubation time at 95 °C followed by 40 cycles of 95 °C for 15 s and 60 °C for 60 s in a Mastercycler ep realplex (Eppendorf, Hamburg, Germany). Relative amount of mRNA was normalized to the β-actin content for each sample.

### 4.12. Statistical Analysis

The data were analyzed using SPSS 20. First, the normality of data distribution was tested by Kolmogorov–Smirnov or Shapiro–Wilk test. The two-tailed Student’s *t*-test or nonparametric Mann–Whitney Rank Sum test was used. All data in this study were expressed as the mean + SD. The statistical significance was assumed at * *p* < 0.05, ** *p* < 0.01; *** *p* < 0.001.

## 5. Conclusions

In conclusion, we report that Pt(*S*-pr-thiosal)2, achieves a very convincing antitumor effect in murine B cell leukemia lymphoma cells, BCL1, treated in vitro, but more importantly in vivo. This effect was achieved by the modulation of the activity of two signaling pathways, STAT3 and NF-κB, and the consecutive altering of the expression of several molecules, p27, c-Myc, and the ratio of pro- and anti-apoptotic proteins, ultimately resulting in the induction of caspase-3 dependent apoptosis and attenuation of cancer stemness. Keeping in mind that these results are obtained using only one murine cell line, there is a necessity for further testing of the Pt(*S*-pr-thiosal)2 complex as a possible new anticancer agent or auxiliary drug for chemotherapy-resistant CLL, and especially for Richter transformation of CLL to B lymphomas which are treated by combined regimens that include platinum-based drugs.
